# Adjuvant radiotherapy and chemotherapy improve survival in patients with pancreatic adenocarcinoma receiving surgery: adjuvant chemotherapy alone is insufficient in the era of intensity modulation radiation therapy

**DOI:** 10.1002/cam4.1479

**Published:** 2018-04-17

**Authors:** Mao‐Chih Hsieh, Wei‐Wen Chang, Hsin‐Hsien Yu, Chang‐Yun Lu, Chia‐Lun Chang, Jyh‐Ming Chow, Shee‐Uan Chen, Yunfeng Cheng, Szu‐Yuan Wu

**Affiliations:** ^1^ Department of General Surgery Wan Fang Hospital Taipei Medical University Taipei Taiwan; ^2^ Department of Hemato‐Oncology Wan Fang Hospital Taipei Medical University Taipei Taiwan; ^3^ Department of Obstetrics and Gynecology National Taiwan University Hospital Taipei Taiwan; ^4^ Department of Hematology Zhongshan Hospital Fudan University Shanghai China; ^5^ Department of Hematology Zhongshan Hospital Qingpu Branch Fudan Universiy Shanghai China; ^6^ Institute of Clinical Science Zhongshan Hospital Fudan University Shanghai China; ^7^ Shanghai Institute of Clinical Bioinformatics Fudan University Center for Clinical Bioinformatics Shanghai China; ^8^ Department of Radiation Oncology Wan Fang Hospital Taipei Medical University Taipei Taiwan; ^9^ Department of Internal Medicine School of Medicine College of Medicine Taipei Medical University Taipei Taiwan

**Keywords:** Adjuvant chemotherapy alone, concurrent chemoradiotherapy, pancreatic adenocarcinoma, sequential chemoradiotherapy, survival

## Abstract

In the era of intensity modulation radiation therapy (IMRT), no prospective randomized trial has evaluated the efficacy of adjuvant therapies such as adjuvant concurrent chemoradiotherapy (CCRT), adjuvant sequential chemotherapy and radiotherapy (CT‐RT), and adjuvant CT alone in resectable pancreatic adenocarcinoma (PA). Through propensity score matching, we designed a nationwide, population‐based, head‐to‐head cohort study to determine the effects of dissimilar adjuvant treatments on resectable PA. We minimized the confounding of various adjuvant treatment outcomes among the following resectable PA groups of patients from the Taiwan Cancer Registry database: group 1, adjuvant CCRT; group 2, adjuvant sequential CT‐RT; and group 3, adjuvant CT alone. All the studied techniques are IMRTs. The matching process yielded a final cohort of 588 patients (196, 196, and 196 patients in groups 1, 2, and 3, respectively). In both univariate and multivariate Cox regression analyses, adjusted hazard ratios (aHRs; 95% confidence interval [CI]) of death derived for the adjuvant CCRT and adjuvant sequential CT‐RT cohorts compared with the adjuvant CT alone cohort were 0.398 (0.314–0.504) and 0.307 (0.235–0.402), respectively. A combination of adjuvant IMRT and CT for resectable PA treatment improves survival to a greater extent than does adjuvant CT alone.

## Introduction

Pancreatic adenocarcinoma (PA) is a highly lethal malignancy 1, 2. It is the fourth leading cause of cancer‐related death in the United States (US) and second only to colorectal cancer as a cause of digestive cancer‐related death 2. In Taiwan, PA is the fifth leading cause of cancer‐related death in women and eighth leading cause of cancer‐related death in men 3. Surgical resection is the only potentially curative treatment 4. However, the prognosis is poor even after complete resection 1, and the 5‐year survival after margin‐negative surgery remains dismal 1. High rates of distant metastasis (more than 80%) and local recurrence (more than 20%) are observed after surgery alone, and adjuvant chemotherapy (CT), radiotherapy (RT), and combined approaches have been used following surgical resection in an effort to improve cure rates 5, 6, 7, 8, 9, 10. Adjuvant CT alone is considered the standard of care in Europe 9, 11. However, the survival periods in patients receiving surgery treated with adjuvant CT alone for resectable PA have been dismal in previous studies 9, 11. Until now, no consensus has been achieved on the optimal adjuvant therapy strategy for the combination of RT and adjuvant CT, and the approach differs in the United States and elsewhere 9, 11, 12. Although adjuvant CT has been demonstrated to improve overall survival (OS), the benefits of adjuvant RT remain controversial 9, 11. New precise RT techniques such as intensity modulation radiation therapy (IMRT) could be less toxic to normal tissue, could involve dose escalation to tumors, and might provide evidence regarding whether a combination of RT and adjuvant CT improves survival 13, 14, 15, 16.

Adjuvant treatment options for resectable PA in patients receiving surgery have not been well established. In the era of IMRT, these adjuvant options include adjuvant concurrent chemoradiotherapy (CCRT) with IMRT, adjuvant sequential CT and RT (CT‐RT) with IMRT, and adjuvant CT alone with fluoropyrimidine or gemcitabine‐based CT regimens. No randomized trial has directly compared these approaches. Using propensity score matching with the Mahalanobis metric (PSM‐MM), we conducted a nationwide, population‐based, cohort study to investigate the effectiveness of different adjuvant treatments in patients with resectable PA receiving surgery.

## Patients and Methods

### Database

Using the data from the Taiwan Cancer Registry database, we enrolled patients who received a diagnosis of resectable PA and underwent surgery between 1 January 2006 and 31 December 2014. The index date was the date of adjuvant therapy. The follow‐up duration was from the index date to 31 December 2016. Our protocols were reviewed and approved by the Institutional Review Board of Taipei Medical University. The Cancer Registry database of the Collaboration Center of Health Information Application contains detailed cancer‐related information on clinical stages, RT doses, RT techniques, and CT regimens used 17, 18, 19, 20, 21, 22, 23, 24.

### Selection of study participants

The diagnoses of the enrolled patients were confirmed on the basis of their pathological data, and patients who had received a new diagnosis of resectable PA were confirmed to have no other cancer or distant metastasis. The inclusion criteria were a diagnosis of resectable PA with a history of surgical resection for PA, age ≥20 years, and American Joint Committee on Cancer (AJCC) clinical cancer stages I‐IV (without metastasis). All RT techniques were IMRTs. The exclusion criteria were a history of cancer before PA diagnosis, distant metastasis, missing sex data, age <20 years, unclear pathological staging, and nonadenocarcinoma histology. PA was defined with the pathological confirmation that was recorded in the Cancer Registry database. In addition, we excluded patients with PA who did not receive any treatments, in those who received sequential CT and RT or CCRT after PA diagnosis did not use fluoropyrimidine or gemcitabine‐based CT regimens, did not use IMRT techniques, received IMRT alone, or underwent adjuvant therapy for more than 12 weeks after surgery. All patients received curative‐intent surgery instead of palliative surgery in our study. Because of no consensus of adjuvant therapy in the setting of patients receiving curative‐intent surgery, the decision of adjuvant treatments could be made by the decision from the tumor board at each hospital in Taiwan. The national cancer registry in Taiwan has survey and validates the rationales of treatments guidelines from hospitals every year. The details of RT were deficient including heterogeneity, dose volume coverage of this target, and normal organ protection. Only treatment modalities, total dose, and treatment interval were recorded in the Cancer registry data. However, national cancer registry in Taiwan has survey and validates the standards for RT based on Quantitative Analyses of Normal Tissue Effects in the Clinic (QUANTEC) or RTOG Radiation Dose Constraints every year. There were 45.12% patients undergoing curative‐intent surgery for pancreatic cancer underwent any adjuvant therapy in our study. Finally, we enrolled patients with resectable PA receiving surgery and categorized them into the following groups on the basis of adjuvant treatment modality to compare their outcomes: group 1, those receiving adjuvant CCRT; group 2, those receiving adjuvant sequential CT‐RT; and group 3, those receiving adjuvant CT alone. The median total dose and fraction size of RT were 50 and 2 Gy per fraction in groups 1 and 2, respectively. Comorbidities were scored using the Charlson comorbidity index (CCI) 19, 25. Only comorbidities observed 6 months before the index date were included; comorbid conditions were identified and included according to the main International Classification of Diseases, Ninth Revision, Clinical Modification (ICD‐9‐CM) diagnosis codes for the first admission or more than two repeated main diagnosis codes for visits to the outpatient department.

### Exposure assessment

The primary outcome of interest in this study was death in different adjuvant treatments. To reduce the effects of potential confounding factors on the comparison of different adjuvant therapy outcomes between groups, PSM was calculated to create well‐balanced groups. The PS was estimated using a multivariable logistic regression model, with treatment groups as dependent variables and potential confounders as covariates. The following confounders were included in the PSM‐MM: age, sex, CT‐based regimens, AJCC pathological stages, CCI, marginal status, cumulative CT dose, and IMRT dose. All patients with resectable PA in the adjuvant CCRT group were matched at a ratio of 1:1 to patients in the adjuvant sequential CT‐RT and in the adjuvant CT alone groups, according to the PSM, using the global optimum method 26. Multivariate Cox regression analysis produced hazard ratios (HRs), which are relevant for determining whether factors such as different adjuvant therapies, age, sex, CT‐based regimens, AJCC pathological stages, CCI, margin status, cumulative CT dose, and IMRT dose are significant independent predictors. The independent predictors were controlled in the analysis, and the endpoint was the mortality rate in the treatment groups, with group 3 (adjuvant CT alone) serving as the control arm.

### Statistical analysis

The cumulative incidence of death was estimated using the Cox proportional hazards model curves for OS in patients undergoing various adjuvant treatments. After adjustment for confounders, the Cox proportional method was used to model the time from the index date to all‐cause mortality in patients undergoing the adjuvant treatments. In the multivariate analysis, HRs were adjusted for age, sex, CT‐based regimens, AJCC pathological stages, CCI, margin status, cumulative CT dose, and IMRT dose. All analyses were performed using SAS (version 9.3; SAS, Cary, NC). Two‐tailed *P *< 0.05 was considered statistically significant.

The cumulative incidence of death was estimated using the inverse probability of treatment weighting (IPTW)‐adjusted Kaplan–Meier method, and differences among adjuvant treatment modalities were determined using the log‐rank test. After adjustment for confounders such as age, sex, CT‐based regimens, AJCC pathological stages, CCI, margin status, cumulative CT dose, and IMRT dose, the Cox proportional hazards method was used to model the time from the index date to death among patients receiving different adjuvant treatments. Stratified analyses were performed using the IPTW‐adjusted Kaplan–Meier method to evaluate the risk of death associated with different treatment modalities. A two‐tailed *P* value of <0.05 was considered statistically significant.

## Results

The matching process yielded a final cohort of 588 patients (196, 196, and 196 patients in groups 1, 2, and 3, respectively) who were eligible for further analyses; their characteristics are summarized in Table 1. The age, sex, CT‐based regimens, AJCC pathological stages, CCI, marginal status, cumulative CT dose, and IMRT dose were balanced among the three groups (Table 1). The follow‐up duration was not matched in the analysis because survival time was inconsistent in different treatment groups (Table 1). In all, 84.69% of the patients received a gemcitabine‐based CT regimen in the adjuvant CCRT, adjuvant sequential CT‐RT, and adjuvant CT alone groups.

**Table 1:** Characteristics of patients with resectable pancreatic adenocarcinoma who received surgery and different adjuvant treatments with their propensity score‐matched cohort

	Total	Adjuvant CCRT		Adjuvant CT‐RT		Only adjuvant CT		*P* values
		*N*	%	*N*	%	*N*	%	
Observation	588	196		196		196		
Sex								
Female	225	75	38.27	76	38.78	74	37.76	0.9786
Male	363	121	61.73	120	61.22	122	62.24	
Age (years)								
<45	51	17	8.67	18	9.18	16	8.16	0.8079
45–55	91	37	18.88	27	13.78	27	13.78	
55–65	208	66	33.67	70	35.71	72	36.73	
65–75	187	65	33.16	63	32.14	59	30.10	
≥75	51	11	5.61	18	9.18	22	11.22	
Median (IQR)	62 (14)	61 (14)		62 (14.5)		62 (14.5)		0.8941
CCI score								
0	176	64	32.65	59	30.10	53	27.04	0.8302
1	222	77	39.29	68	34.69	77	39.29	
2	121	33	16.84	44	22.45	44	22.45	
3	47	16	8.16	16	8.16	15	7.65	
≥4	22	6	3.06	9	4.59	7	3.57	
Median (IQR)	1 (2)	1 (2)		1 (2)		1 (2)		1.0000
Margin status								
Positive	132	45	22.96	44	22.45	43	21.94	0.8648
Negative	456	151	77.04	152	77.55	153	78.06	
Pathologic AJCC stage								
Stage I–IIA	428	143	72.96	141	71.94	144	73.47	0.8834
Stage IIB–III	160	53	27.04	55	28.06	52	26.53	
Total CT cumulative dose								
Gemcitabine (mg/m^2^)	*N *=* *498	*N *=* *166		*N *=* *166		*N *=* *16		
Median (IQR)	13,200 (7400)	13,168 (6328)		13,200 (8920)		13,400 (7350)		0.8091
Fluoropyrimidine (mg/m^2^)	*N *=* *90	*N *=* *30		*N *=* *30		*N *=* *30		
Median (IQR)	18,750 (3700)	18,700 (3400)		18,730 (3432)		18,800 (3146)		0.8076
IMRT dose								
Median total dose (Gy)	50.00	50.00		50.00		–	–	<0.0001
IQR	10.00	10.00		10.00		–	–	
Median fraction size	2.00	2.00		2.00				
Follow‐up duration (days)								
Median (IQR)	331.50 (356.00)	354.50 (447.5)		403 (364)		244 (257.5)		<0.0001
Death								
No	124	41	20.92	52	26.53	31	15.82	0.0340
Yes	464	155	79.08	144	73.47	165	84.18	

RT, radiotherapy; CT, chemotherapy; CCRT, concurrent chemoradiotherapy; CCI, Charlson comorbidity index; IQR, interquartile range; AJCC, American Joint Committee on Cancer; IMRT, intensity modulation radiation therapy.

According to the multivariate Cox regression analysis, different adjuvant treatments were significant independent predictors of OS (Table 2). Both univariate and multivariate Cox regression analyses indicated that adjuvant CCRT and adjuvant sequential CT‐RT were significant independent prognostic risk factors for a more favorable OS. In univariate Cox regression analyses, HRs (95% confidence interval [CI]) derived for the adjuvant CCRT and adjuvant sequential CT‐RT cohorts compared with the adjuvant CT alone cohort were 0.433 (0.345–0.544) and 0.319 (0.246–0.413), respectively. In multivariate Cox regression analyses, adjusted HRs (aHRs; 95% CI) derived for the adjuvant CCRT and adjuvant sequential CT‐RT cohorts compared with the adjuvant CT alone cohort were 0.398 (0.314–0.504) and 0.307 (0.235–0.402), respectively.

**Table 2:** Cox proportional hazard regression analysis of the risk of death among patients with resectable pancreatic adenocarcinoma receiving surgery

	Univariate analysis			Multivariate analysis		
	HR	*P* values	95% CI	aHR	*P* values	95% CI
Treatment						
Adj. CT (ref.)	1.000		–	1.000		–
Adj. CCRT	0.433	<0.0001	0.345–0.544	0.398	<0.0001	0.314–0.504
Adj. CT‐RT	0.319	<0.0001	0.246–0.413	0.307	<0.0001	0.235–0.402
Sex						
Female (ref.)	1.000		–	1.000		–
Male	0.932	0.8811	0.370–2.350	0.889	0.7926	0.369–2.142
Age (years)						
<45 (ref.)	1.000		–	1.000		–
45–55	0.843	0.7267	0.324–2.196	1.026	0.9687	0.287–3.666
55–65	0.987	0.9798	0.373–2.616	0.897	0.8611	0.266–3.023
65–75	1.182	0.7468	0.428–3.262	1.275	0.7006	0.369–4.407
≥75	1.342	0.6480	0.380–4.745	1.248	0.7685	0.285–5.462
CCI score						
0 (ref.)	1.000		–	1.000		–
1	0.856	0.5162	0.534–1.370	0.640	0.3719	0.394–1.041
2	0.902	0.4971	0.553–1.099	0.929	0.4037	0.455–1.197
3	0.816	0.2112	0.685–1.246	0.819	0.1121	0.640–1.357
≥4	1.089	0.1639	0.103–1.470	1.141	0.1668	0.103–1.103
Margin status						
Negative (ref.)	1.000		–			–
Positive	1.034	0.7993	0.674–1.163	1.012	0.4237	0.842–1.142
Adjuvant CT‐based regimen						
Fluoropyrimidine (ref.)	1.000		–			–
Gemcitabine	0.646	0.0758	0.399–1.046	0.997	0.8588	0.964–1.031
Pathologic AJCC stage						
Stage I–IIA (ref.)	1.000		–			–
Stage IIB–III	1.304	0.6182	0.459–3.709	1.261	0.6397	0.478–3.330

All the aforementioned variables were used in the multivariate analysis.

CCRT, concurrent chemoradiotherapy; CCI, Charlson comorbidity index; CI, confidence interval; aHR, adjusted hazard ratio; RT, radiotherapy; CT, chemotherapy; AJCC, American Joint Committee on Cancer; Ref, reference group.

In different CT‐based regimens, the multivariate Cox regression analysis revealed that adjuvant CCRT and adjuvant sequential CT‐RT were still more significant independent prognostic risk factors for a more favorable OS than adjuvant gemcitabine or fluoropyrimidine‐based CT (Tables S1 and S2). The aHRs derived for the adjuvant CCRT and adjuvant sequential CT‐RT cohorts compared with the adjuvant CT alone cohort were 0.470 (0.361–0.613) and 0.415 (0.318–0.542), respectively, in adjuvant gemcitabine‐based CT. The survival remained significantly poorer in the adjuvant gemcitabine‐based CT alone cohort (Table S1). In adjuvant fluoropyrimidine‐based CT, both univariate and multivariate Cox regression analyses indicated that adjuvant CCRT and adjuvant sequential CT‐RT were significant independent prognostic risk factors for a more favorable OS. The aHRs (95% CI) derived for the adjuvant CCRT and adjuvant sequential CT‐RT cohorts compared with the adjuvant fluoropyrimidine‐based CT alone cohort were 0.362 (0.156–0.838) and 0.317 (0.146–0.688), respectively (Table S2).

**Figure 1:**
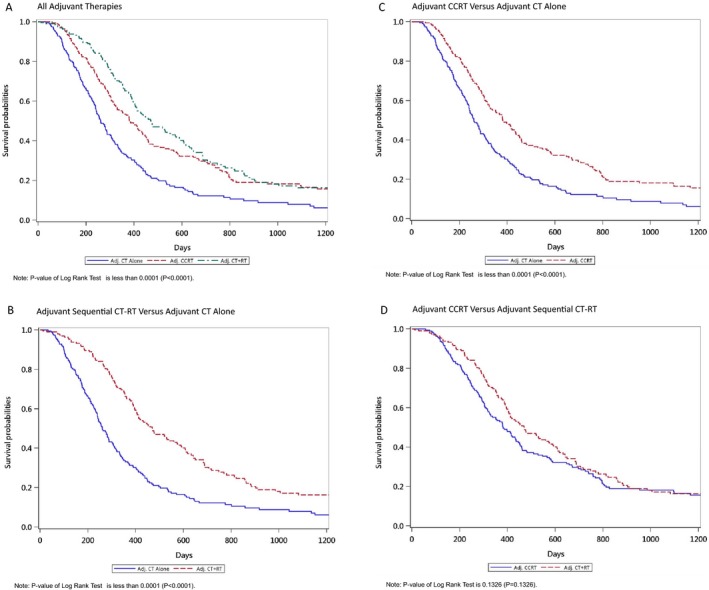
Estimates of overall survival in patients with resectable pancreatic adenocarcinoma who underwent surgery and received different adjuvant therapies, as obtained using the IPTW‐adjusted Kaplan–Meier method. (A) all adjuvant therapies; (B) adjuvant CCRT versus adjuvant CT alone; (C) adjuvant sequential CT‐RT versus adjuvant CT alone; (D) adjuvant CCRT versus adjuvant sequential CT‐RT.

The estimates of the OS in the study patients, obtained using the IPTW‐adjusted Kaplan–Meier method, were used to analyze the risk of death associated with the various adjuvant therapies (Fig. 1A–D). To investigate the risk of death after adjuvant treatment, adjuvant CT alone was used as the control (Fig. 1A). After IPTW adjustment for age, sex, CT‐based regimens, AJCC pathological stages, CCI, margin status, cumulative CT dose, and IMRT dose, results indicated that log‐rank *P *< 0.001 for the cumulative incidence of death (Fig. 1A–C). The survival rate in the adjuvant CCRT or adjuvant sequential CT‐RT groups was superior to that of the adjuvant CT alone group; log‐rank *P *<* *0.001 (Fig. 1A–C). The highest cumulative incidence of death was observed in the adjuvant CT alone group. A comparison between adjuvant CCRT and adjuvant sequential CT‐RT groups yielded log‐rank *P *=* *0.1428 after IPTW adjustment (Fig. 1D).

## Discussion

Tumor stage is the most critical prognostic factor 27. Another crucial prognostic factor for patients with resected PA is nodal status 1, 28. Five‐year survival after surgery is only approximately 10% for lymph node‐positive disease, whereas it is approximately 30% for lymph node‐negative disease 1, 28. In addition to tumor stage and nodal status, the status of the surgical margins (positive or negative) influences prognosis after resection 29, 30, 31, 32. To match these major covariates, we used PSM with stage I‐IIA and stage IIB‐III by including lymph nodal status (nodal positives were included in stage IIB, AJCC 7th edition) in the analysis (Table 1). Margin status was also considered in the PSM. Moreover, age, sex, RT dose, cumulative CT dose, IMRT dose, and CCI scores were included in the PSM analysis.

A rationale for adding RT to CT is provided by the failure pattern following surgical resection alone. In an autopsy series for PA, 15% of those who had surgery alone for stage I or II disease had a local recurrence in the pancreatic bed alone, whereas 65% had both locally recurrent and metastatic disease 33. The local control benefit of adding RT can be most effectively illustrated using data from the Groupe Cooperateur Multidisciplinaire en Oncologie trial of adjuvant gemcitabine‐based CT alone versus adjuvant gemcitabine‐based CCRT 6. The rate of local recurrence alone at first progression in the adjuvant CCRT group was notably lower (11% vs. 24%), as was the rate of simultaneous local and distant progression (13% vs. 20%) 6. However, not all studies have demonstrated improvements in local control with the use of combined modality therapy 9, 11. Furthermore, randomized trials and meta‐analyses have failed to confirm a survival benefit from adjuvant RT 34. The benefits of adjuvant RT with and without adjuvant CT alone were addressed in a 2013 network meta‐analysis of nine randomized trials that compared five distinct adjuvant strategies (observation alone, adjuvant fluoropyrimidine‐based CT alone, gemcitabine‐based CT alone, adjuvant CCRT, and adjuvant sequential CT‐RT) 34. To optimize data extrapolation, the authors used Bayesian network meta‐analysis to compare adjuvant treatments indirectly when no direct comparator trial existed 34. A statistically significant survival benefit for adjuvant CCRT was not demonstrated 34. However, the wide range of CIs reflects a lack of precision in these estimates 34. Thus, it is difficult to draw any meaningful conclusions from these data 34.

The European Study for Pancreatic Cancer (ESPAC)‐1 conducted three separate randomization studies (2 × 2, with or without adjuvant CT and with or without CCRT) to determine the roles of various adjuvant therapy options 8, 9. A combined analysis revealed no survival benefit from adjuvant CCRT with the two‐dimensional conventional technique, but significant benefit from adjuvant CT alone 8, 9. Further analysis of only the patients from the 2 × 2 study revealed a survival benefit associated with adjuvant CT alone and a deleterious effect of the combination of RT and CT 8, 9. The ESPAC‐1 trial, an ambitious trial sponsored by European investigators, was initially conducted by randomizing patients into a 2 × 2 factorial design to compare the relative benefits of adjuvant CT alone, adjuvant CCRT, or adjuvant CCRT followed by consolidative CT with observation alone 8, 9. However, the fear of poor accrual led the investigators to permit the clinician to choose from this or two other randomization schemes 8, 9. The final results have been presented in two separate publications, one that pooled the results from the three parallel randomized trials 8 and a later report that focused on the 289 patients randomized to the four‐arm study 9. The ESPAC‐1 trial has been widely criticized for its methodology 35, 36.

In the 1990s, the European Organization for Research and Treatment of Cancer (EORTC) 40891 attempted to confirm the GITSG 9173 results 11, 37. It randomized patients to the adjuvant CT‐RT and no adjuvant treatment groups. The RT schedule was the same as that of GITSG 9173 (two‐dimensional conventional RT technique and insufficient RT dose, 40 Gy); the adjuvant CT schedule was concurrent fluoropyrimidine only. EORTC 40891 also included patients with periampullary cancers (45%), who have a significantly more favorable prognosis; the population was heterogeneous and did not entirely comprise patients with PA 11. Moreover, no benefit for OS was observed when the RT group was added in EORTC 40891 11. An exploratory subset analysis also revealed no benefit for the PA subset 11. Based on the combined results of EORTC 40891 and ESPAC‐1, adjuvant CCRT is not indicated in Europe 9, 11.

Adjuvant gemcitabine‐based CT alone is the preferred agent for adjuvant CT compared with adjuvant fluoropyrimidine‐based CT alone, due to its greater tolerability 38. Preliminary data support the tolerability and favorable short‐term outcomes of regimens that use gemcitabine as a radiation sensitizer 6, 39, 40, 41; however, no trials have compared this approach with adjuvant CT‐RT using fluoropyrimidine as the radiation sensitizer, at least in the postoperative setting. In our study, we also estimated the survival benefits of adjuvant CT‐RT using fluoropyrimidine as the radiation sensitizer (Table S2). A German trial (CONKO‐001) demonstrated a significant survival advantage associated with adjuvant gemcitabine‐based CT alone over observation alone 5, 42; no benefit was observed in a Japanese trial of adjuvant fluoropyrimidine‐based CT alone 43. ESPAC‐3 trial compared observation alone to adjuvant fluoropyrimidine‐based CT alone (ESPAC‐1) and to adjuvant gemcitabine‐based CT alone (CONKO‐001) 38. No significant differences in survival between adjuvant fluoropyrimidine and gemcitabine‐based CT alone were observed 38. In our study, we stratified various CT‐based treatments in our analysis (Tables S1 and S2). After PSM‐MM analysis, adjuvant CCRT or adjuvant sequential CT‐RT could improve OS and reduce the death risk to a higher extent than adjuvant CT alone instead of adjuvant fluoropyrimidine or gemcitabine‐based CT alone (Tables S1 and S2). The outcomes of another two retrospective cohort studies without detail RT techniques were compatible with ours which demonstrated addition of RT to adjuvant CT is associated with improved OS in resected PA 44, 45. The RTOG and now NRG Oncology in the USA have had an intergroup study (RTOG 0848) open for 9 years addressing this question in a prospective randomized fashion, but still open to accrual. This is the leading study to demonstrate that a combination of IMRT and adjuvant fluoropyrimidine or gemcitabine‐based CT could improve survival in patients with resectable PA receiving surgery.

Usually, therapeutic policy might be changed due to improvement in RT techniques 17. Higher dose RT approaches that use IMRT with or without CT may be associated with a higher local control, less toxicity, lower RT dose escalation, reduction in normal tissue RT dose, and possibly prolonged survival in patients with PA 13, 14, 15, 16. This is the first study to investigate whether IMRT in the adjuvant therapy setting improves survival. Studies have mostly used conventional RT techniques with insufficient RT doses 9, 11. The toxicity of conventional RT might be too high to provide survival benefits in these randomized trials or meta‐analyses^9^, ^11^, ^34^; however, some retrospective studies have shown that the addition of adjuvant RT might be beneficial for survival in patients with PA receiving surgery 44, 46, 47, 48. In our study, the fraction size and total RT dose were homogenous and compatible with those in previous small and retrospective studies with IMRT for PA 13, 14, 15, 16. This is the first and largest series with head‐to‐head PSM‐MM to demonstrate that adding RT to CT with IMRT techniques improves the OS to a greater extent compared with adjuvant CT alone in patients with resectable PA receiving surgery.

A randomized controlled trial is considered an ideal evaluation technique for estimating treatment effects because successful randomization minimizes or entirely avoids measurable and unmeasurable differences between the treatment and control groups, leaving only one variable (*i.e*., assignment to the treatment or control group), which would be likely to cause differences in the observed outcomes. However, randomization is often not feasible or permissible in rare malignancies, such as in patients with resectable PA receiving surgery because only 15–20% of the cases are potentially resectable at presentation 1. To resolve the problem of selection bias and small sample sizes for rare malignancies, we designed a PSM‐MM, nationwide, population‐based, cohort study. Interval matching using the Mahalanobis distance is a promising alternative tool for reducing selection bias in deriving a causal inference from observational studies and is particularly useful in secondary data analysis of national databases, such as the Centers for Medicare and Medicaid Services 49. We used the adjuvant CCRT cohort as the case arm and the adjuvant CT alone cohort as the control arm. We matched the patients in the adjuvant CCRT cohort to the patients in the adjuvant sequential CT‐RT and to the adjuvant CT alone cohorts at a ratio of 1:1 according to the PSM using the interval matching method 49. In our study, 37 patients did not exhibit a match; in the case arm, 14.03% of patients did not exhibit a match. The percentage of the patients that did not exhibit a match was small 50. As listed in Table 1, all *P* values were between 0.8 and 1, which indicate that our matching results were satisfactory and the confounding factors could be effectively controlled 50. The PSM‐MM joint consideration of PSM and multivariate analysis enables the assessment of the robustness of estimates 50. The sensitivity analysis of the PSM‐MM technique is crucial because PSM‐MM considerably reduces the deviation in estimates 50. The PSM‐MM technique balances the distributions of observed covariates between treatment conditions and thus approximates a situation that is normally achieved through randomization 49, 50.

The residual confounding was inadequate to cause residual imbalance (Tables 2, S1 and S2). The results of our matching were satisfactory. Thus, in univariate or multivariate analyses, HRs were not significant for age, sex, CT‐based regimens, AJCC pathological stages, CCI, margin status, and IMRT dose (Tables 2, S1 and S2). Only mortality exerted an effect on various adjuvant treatments for resectable PA. As shown in Table 2, the addition of IMRT to CT could reduce the risk of death, irrespective of whether patients received adjuvant CCRT or adjuvant sequential CT‐RT. As shown in Figure 1, the IPTW adjustment revealed that the log‐rank *P* value of the cumulative incidence of death in different adjuvant therapies was <0.001. The pessimal OS curve was obtained for the adjuvant CT alone cohort. Compared with adjuvant CT alone, the combination of RT and CT resulted in a more favorable OS (Fig. 1B and C). According to our findings, adjuvant CT alone should not be suggested for patients with resectable PA receiving surgery in the era of IMRT. Our results demonstrated the importance and value of adjuvant IMRT in the treatment of patients with resectable PA receiving surgery.

The strengths of this study are its large sample size and the homogeneity of the PA population with IMRT. Our current study had homogenous pathology (all adenocarcinoma), similar treatment modality with IMRT, and homogenous RT dose. Most major covariates such as margin status, pathological stages, RT dose, RT fraction size, age, sex, cumulative CT dose, and CCI scores were considered in the PSM analysis. This is the first and largest head‐to‐head PSM‐MM study to estimate the effect of adding IMRT to adjuvant CT alone. According to our findings, the addition of IMRT to adjuvant CT alone for resectable PA treatment is more beneficial than adjuvant CT alone, irrespective of whether adjuvant CCRT or adjuvant sequential CT‐RT is used (Table 2 and Fig. 1A–D). Considering various CT‐based regimens, the addition of IMRT to both adjuvant fluoropyrimidine and gemcitabine‐based CT improved the OS (Tables S1 and S2). These findings should be considered in future clinical studies.

This study has some limitations. First, the toxicity induced by the various treatments could not be determined; therefore, treatment‐related mortality estimates may have been biased. However, a previous study demonstrated more complications and a higher mortality in CCRT than in CT alone 51. The benefits of an improved survival rate after CCRT therapy in the current study may be underestimated. Second, because all patients with resectable PA were enrolled from an Asian population, the corresponding ethnic susceptibility remains unclear; hence, our results should be cautiously extrapolated to non‐Asian populations. Third, the diagnoses of all comorbid conditions were based on ICD‐9‐CM codes. Nevertheless, the Taiwan Cancer Registry Administration randomly reviews charts and interviews patients to verify the accuracy of the diagnoses, and hospitals with outlier chargers or practices may be audited and subsequently be heavily penalized if malpractice or discrepancies are identified. Therefore, to obtain crucial information on population specificity and disease occurrence, a large‐scale randomized trial comparing carefully selected patients undergoing suitable treatments is essential. Fourth, persistent postoperative elevations of the serum tumor marker CA 19‐9 are associated with poor long‐term prognosis 52; however, data on CA 19‐9 are not available in the current database. Nevertheless, CA 19‐9 levels are prognostic and not predictive of benefit from adjuvant therapy 53. Some experts suggest not using postoperative CA 19‐9 levels to determine whether to administer adjuvant therapy outside the context of a clinical trial 53. Fifth, although surgical procedures were different between periampullary or distal pancreatic in location, the location of PA was unavailable in the database. However, the postoperative pathologic risk factors such as margin, N stage, and T stage were available in the study. Finally, the Cancer Registry database does not contain information on dietary habits, socioeconomic status, performance status, or body mass index, all of which may be risk factors for mortality. However, considering the magnitude and statistical significance of the observed effects in this study, these limitations are unlikely to affect the conclusions.

## Conclusions

The combination of adjuvant IMRT and CT for treatment of resectable PA in patients receiving surgery improves the survival to a greater extent than adjuvant CT alone. Combining IMRT with both adjuvant fluoropyrimidine and gemcitabine‐based CT improved the OS.

## Ethics Approval and Consent

Our protocols were reviewed and approved by the Institutional Review Board of Taipei Medical University (TMU‐JIRB No. 201402018).

## Conflict of Interest

The authors have no potential conflict of interests to declare. The data sets supporting the study conclusions are included within the manuscript.

## Supporting information


**Table S1.** Cox proportional hazard regression analysis of the risk of death among patients with resectable pancreatic adenocarcinoma receiving surgery with adjuvant gemcitabine‐based CT.

**Table S2.** Cox Proportional hazard regression analysis of the risk of death among patients with resectable pancreatic adenocarcinoma receiving surgery with adjuvant fluoropyrimidine‐based CT.
