# Supplementary material for: Adjuvant radiotherapy and chemotherapy improve survival in patients with pancreatic adenocarcinoma receiving surgery: adjuvant chemotherapy alone is insufficient in the era of intensity modulation radiation therapy
Source: Cancer Med. 2018 Apr 17;7(6):2328–38. doi: 10.1002/cam4.1479 (PMC6010773; doi:10.1002/cam4.1479)
Supplement: Supplementary file 1 — Table S1. Cox proportional hazard regression analysis of the risk of death among patients with resectable pancreatic adenocarcinoma receiving surgery with adjuvant gemcitabine‐based CT. [file CAM4-7-2328-s001.docx]

**Supplemental Table 1.** Cox Proportional Hazard Regression Analysis of the Risk of Death Among Patients with Resectable Pancreatic Adenocarcinoma Receiving Surgery with Adjuvant Gemcitabine-Based CT

|  |  | | | | **Univariate analysis** | | |  | | **Multivariate analysis** | | |
| --- | --- | --- | --- | --- | --- | --- | --- | --- | --- | --- | --- | --- |
|  | **HR** | | ***P* value** | | | **95% CI** | | **aHR** | | | ***P* value** | **95% CI** |
| **Treatment** |  | |  | | |  | |  | | |  |  |
| **Adj. CT (ref.)** | 1.000 | |  | | | - | | 1.000 | | |  | - |
| **Adj. CCRT** | 0.478 | | <.0001 | | | (0.369-0.619) | | 0.470 | | | <.0001 | (0.361-0.613) |
| **Adj. CT-RT** | 0.415 | | <.0001 | | | (0.318-0.541) | | 0.415 | | | <.0001 | (0.318-0.542) |
| **Sex** |  | |  | | |  | |  | | |  |  |
| **Female (ref.)** | 1.000 | |  | | | - | | 1.000 | | |  | - |
| **Male** | 0.889 | | 0.7926 | | | (0.369-2.142) | | 0.856 | | | 0.7420 | (0.339-2.161) |
| **Age (y)** |  | |  | | |  | |  | | |  |  |
| **<45 (ref.)** | 1.000 | |  | | | - | | 1.000 | | |  | - |
| **45-55** | 0.733 | | 0.4892 | | | (0.304-1.767) | | 0.843 | | | 0.7237 | (0.326-2.176) |
| **55-65** | 0.796 | | 0.6470 | | | (0.300-2.112) | | 0.848 | | | 0.7582 | (0.296-2.425) |
| **65-75** | 0.603 | | 0.3320 | | | (0.218-1.674) | | 0.712 | | | 0.5524 | (0.232-2.184) |
| **≥75** | 1.423 | | 0.6347 | | | (0.332-6.099) | | 1.488 | | | 0.6669 | (0.244-9.092) |
| **CCI score** |  | |  | | |  | |  | | |  |  |
| **0 (ref.)** | 1.000 | |  | | | - | | 1.000 | | |  | - |
| **1** | 0.876 | | 0.7161 | | | (0.429-1.790) | | 0.834 | | | 0.6269 | (0.400-1.737) |
| **2** | 0.979 | | 0.9603 | | | (0.417-2.297) | | 0.877 | | | 0.7754 | (0.354-2.168) |
| **3** | 0.614 | | 0.4350 | | | (0.180-2.089) | | 0.671 | | | 0.5642 | (0.173-2.604) |
| **≥4** | 0.839 | | 0.8115 | | | (0.197-3.564) | | 1.209 | | | 0.8183 | (0.239-6.121) |
| **Margin status** | |  | |  | | |  | |  | |  |  |
| **Negative(ref.)** | | 1.000 | |  | | | - | |  | |  | - |
| **Positive** | | 1.068 | | 0.9398 | | | (0.813-1.267) | | 1.081 | | 0.7842 | (0.656-1.280) |
| **Pathologic AJCC stage** | |  | |  | | |  | |  | |  |  |
| **Stage I-IIA(ref.)** | | 1.000 | |  | | | - | |  | |  | - |
| **Stage IIB-III** | | 1.234 | | 0.7849 | | | 0.542-1.998 | | 1.327 | | 0.7561 | 0.604-1.761 |

*All the aforementioned variables were used in multivariate analysis.

CCRT, concurrent chemoradiotherapy; CCI, Charlson comorbidity index; CI, confidence interval; aHR, adjusted hazard ratio; RT, radiotherapy; CT, chemotherapy; AJCC, American Joint Committee on Cancer; Ref, reference group.
