# Supplementary material for: Adjuvant radiotherapy and chemotherapy improve survival in patients with pancreatic adenocarcinoma receiving surgery: adjuvant chemotherapy alone is insufficient in the era of intensity modulation radiation therapy
Source: Cancer Med. 2018 Apr 17;7(6):2328–38. doi: 10.1002/cam4.1479 (PMC6010773; doi:10.1002/cam4.1479)
Supplement: Supplementary file 2 — Table S2. Cox Proportional hazard regression analysis of the risk of death among patients with resectable pancreatic adenocarcinoma receiving surgery with adjuvant fluoropyrimidine‐based CT. [file CAM4-7-2328-s002.docx]

**Supplemental Table 2.** Cox Proportional Hazard Regression Analysis of the Risk of Death Among Patients with Resectable Pancreatic Adenocarcinoma Receiving Surgery with Adjuvant Fluoropyrimidine-Based CT

|  |  | | | | **Univariate analysis** | | |  | **Multivariate analysis** | |
| --- | --- | --- | --- | --- | --- | --- | --- | --- | --- | --- |
|  | **HR** | | ***P* value** | | | **95% CI** | | **aHR** | ***P* value** | **95% CI** |
| **Treatment** |  | |  | | |  | |  |  |  |
| **Adj. CT (ref.)** | 1.000 | |  | | | - | | 1.000 |  | - |
| **Adj. CCRT** | 0.383 | | 0.0154 | | | (0.176-0.832) | | 0.362 | 0.0176 | (0.156-0.838) |
| **Adj. CT-RT** | 0.325 | | 0.0023 | | | (0.158-0.670) | | 0.317 | 0.0037 | (0.146-0.688) |
| **Sex** |  | |  | | |  | |  |  |  |
| **Female (ref.)** | 1.000 | |  | | | - | | 1.000 |  | - |
| **Male** | 1.731 | | 0.3220 | | | (0.584-5.124) | | 1.028 | 0.4780 | (0.953-1.109) |
| **Age (y)** |  | |  | | |  | |  |  |  |
| **<45 (ref.)** | 1.000 | |  | | | - | | 1.000 |  | - |
| **45-55** | 1.847 | | 0.3840 | | | (0.643-1.987) | | 1.458 | 0.5320 | (0.643-2.142) |
| **55-65** | 1.167 | | 0.2337 | | | (0.735-1.482) | | 1.627 | 0.2847 | (0.929-2.336) |
| **65-75** | 1.543 | | 0.7035 | | | (0.703-2.132) | | 1.228 | 0.5520 | (0.881-2.262) |
| **≥75** | 1.613 | | 0.6270 | | | (0.825-1.887) | | 1.789 | 0.6633 | (0.865-2.157) |
| **CCI score** |  | |  | | |  | |  |  |  |
| **0 (ref.)** | 1.000 | |  | | | - | | 1.000 |  | - |
| **1** | 1.157 | | 0.8314 | | | (0.303-2.411) | | 1.539 | 0.5276 | (0.779-2.671) |
| **2** | 1.138 | | 0.8714 | | | (0.239-2.422) | | 1.448 | 0.4392 | (0.659-2.827) |
| **3** | 1.520 | | 0.5634 | | | (0.757-2.770) | | 1.214 | 0.2322 | (0.717-2.887) |
| **≥4** | 1.574 | | 0.6768 | | | (0.422-2.830) | | 1.491 | 0.9118 | (0.234-3.092) |
| **Margin status** | |  | |  | | |  |  |  |  |
| **Negative(ref.)** | | 1.000 | |  | | | - |  |  | - |
| **Positive** | | 1.175 | | 0.8118 | | | (0.312-1.422) | 1.201 | 0.8037 | (0.139-1.626) |
| **Pathologic AJCC stage** | |  | |  | | |  |  |  |  |
| **Stage I-IIA(ref.)** | | 1.000 | |  | | | - |  |  | - |
| **Stage IIB-III** | | 1.754 | | 0.6006 | | | (0.389-2.878) | 1.449 | 0.5962 | (0.326-2.480) |

*All the aforementioned variables were used in multivariate analysis.

CCRT, concurrent chemoradiotherapy; CCI, Charlson comorbidity index; CI, confidence interval; aHR, adjusted hazard ratio; RT, radiotherapy; CT, chemotherapy; AJCC, American Joint Committee on Cancer; Ref, reference group.
